# Report on the Malady at Walcheren

**Published:** 1810-03

**Authors:** J. Borland, W. Lempriere, G. Blane

**Affiliations:** Inspector of Hospitals; Physician to the Forces


*The following Paper, besides the many useful Medical Remarks it contains, we wish to preserve as a valuable Record, in Addition to what may be found in Sir John Pringle's Works.*

REPORT on the prevailing Malady among his Majesty's Forces serving in the Island of Walcheren.

1st.—Nature of the Disease and its Causes.

THE malady prevailing among the British troops in Walcheren, is the endemic fever of marshy countries; the effect of miasmata from a soil the most productive in deleterious exhalations of any, perhaps, in Europe. Its effects, however, are not confined to the British forces, for the natives are annually liable to a similar calamity; and, by the sick returns of the French army for the last seven years, it appears that, at least, one-third of its force has been yearly cut off by the endemic. It has, however, been observed, that, among the highest orders of the inhabitants, and the officers of the British army who have slept in upper apartments, and whose mode of life and comforts are necessarily superior, although they do suffer by the disease, yet the attack altogether is milder and less fatal in its termination; a convincing proof how much good may result from judicious interior economy in an army.

The disease is remittent or intermittent in form, according to circumstances, constantly following a general law of season; appearing among the inhabitants towards the end of summer; becoming more severe in the autumnal months, particularly August and September, declining in October, and nearly ceasing in November.

Among British troops, whose constitutions have not been habituated to the climate, who have been exposed to previous military fatigues, to external wet, to damp, and to crouded accommodation, the disease bears a character of

 greater greater malignancy, corresponding with the degrees or combinations of all these predisposing causes.

The malady is not contagious in itself, but liable to assume that new form of fever wherever ventilation is defective, the patients crouded, or where other local causes of impurity prevail. This has been strikingly proved in some instances, particularly at Flushing, where we found the accomodations too confined and crouded, where the ditches are foul and obstructed, from the consequences of the late siege, and the streets filthy from imperfect police.

The robust young soldier is more prone to the endemic disease than the old, puny, or consumptive; and such men as have been recruited from dry mountainous districts, are more liable to be affected than those from flat and fenny countries. A proof of the power of habit to which we shall hereafter advert.

The tendency to relapse is very great, and the deaths therefrom have been numerous, and sometimes sudden. Perfect recoveries are rare; convalescence never secure; and where the recurrence of fever do not destroy life, they quickly lay foundations for permanent visceral obstructions; rendering a large proportion of the sufferers inefficient for future military purposes.

The soldier who has entirely resisted the epidemic of this year, may be expected to enjoy a good state of health until the succeeding autumn; when, though not exempt from an attack, he will be much less liable than one newly arrived.

But such men as have suffered from the prevailing malady will run the risk of being cut off by the inflammatory disease of the ensuing winter and spring, particularly March; in which month, according to the testimony of the inhabitants, such complaints are more frequent and fatal.

By the sick returns obtained from the inspector's office, for the last four weeks, it appears that, though the number of sick have increased, yet the actual mortality has gradually diminished; and, at our last inspection of the hospitals, we remarked, that the epidemic had assumed an aspect somewhat milder than upon our first arrival; leading us to hope that it is on the decline; notwithstanding, for some weeks to come, attacks may be expected among those who have not yet been affected, and its total extinction not accomplished till the winter has regularly set in; which, in this country, usually takes place in the month of November.

2d.—Present

2d.—Present State of the Sick; and Suggestions for affording immediate Relief to the Hospitals.

The numbers of sick and convalescents in the different hospitals, amount to more than two-thirds of the total force; and, notwithstanding about 1,500 sick have already been sent home by different conveyances from Walcheren alone, the constant occurrence of recent disease, and the numerous relapses, have necessarily kept the hospitals full, even beyond a just proportion; while the tedious recoveries have not permitted the discharges to keep pace with the daily admissions.

To give prompt relief to the existing pressure, to prevent the increased malignancy generated by the effluvia of a crouded hospital, to guard against the production of a contagious fever and dysentery, and to save the lives of many who linger in doubtful convalescence, it is suggested, that six line of battle ships, armed *en flute*, be properly fitted up in England, and be dispatched to receive all the convalescents and cases of fever, that can be removed with safety; each ship may take on board 300, and thus the hospitals on shore be relieved by the abstraction of 1800 persons.

The ships to land the sick at the different ports of Harwich, Deal, Portsmouth, or Plymouth, according to the accommodation those places respectively afford: a proportion of convalescent officers to accompany the sick, in order to keep up military discipline, and to enforce medical regulations. Bedding, medicines, diet, &c. of every description, to be furnished by the men of war, to save the stores so urgently wanted in Walcheren.

The pursers of the ships to keep proper lists of hospital stoppages, to be charged against the agent of the corps to which the sick belong.

The naval surgeons and mates (with the assistance of an army hospital mate, if required), will be adequate to the medical attendance on the passage, and thus the medical staff of the army be reserved for the duties on shore. These ships to return a second or third time, as may be necessary. One of the many advantages attending this scheme is, that each ship might sail singly without incurring the inconveniences and hardships attending the detention for convoy, and of embarking and disembarking great numbers at the same time in fleets of transports.

Independent of this arrangement, for removing sick to England, as an immediate relief to the hospitals, it is also suggested, suggested, that there should be always roomy hospital ships in readiness to receive men, who are in the first stage of convalescence, to prevent the relapses so frequent and so fatal on shore; which ships should proceed to England with a fair wind, as occasion may offer, or according as the head of the medical department in Walcheren may recommend, for it is to be kept in constant recollection, that it is slow recoveries and relapses that cause the principal mortality, and swell the numbers in the hospitals, and that it is the prompt removal alone of convalescents to England, that can insure ultimate recovery.

3d.—Prospective Arrangements for preventing future Sickness, and for arresting the Progress of the Malady when it occurs.

The different Battalions now serving in Walcheren, have suffered lamentably by having been suddenly placed under the influence of the endemic miasmata, at the very worst season of the year, with unavoidably defective accommodation, and means ill suited to meet the unexpected calamity, that the majority are unfit for service, and should be removed to England, retaining only such men as have not been attacked by the disease.

The power of habit in reconciling the human constitution to noxious air has already been alluded to; the greater sickness of strangers, the superior healthiness of the natives, and the testimony of the French on this point, sufficiently confirm this principle; and grounding upon it, we presume to recommend to his Majesty's Government, in case the retention of this island should be determined on, to reinforce the garrison very early in the winter, (say November) in order that the constitutions of the men may in some degree be habituated to the climate before the return of the sickly season, which consists of the months of June, July, August, and September.

The troops should not be oppressed with duty, or enjoy less than four nights in bed: On the evenings of the nights on which they mount guard, an extra allowance of spirits to each man would be essentially beneficial; and when relieved next morning, a comfortable warm breakfast of strong coffee should be in readiness.

The Barracks ought to be of the best description, well guarded from cold and damp, with boarded floors; stoves and flues suitably directed to convey an equal temperature to the remotest corners, to be placed in each room. On no account should ground floors be used as sleeping apartments, ments. The more lofty the buildings the better; for the tenants of the upper stories not only enjoy the best health, but when taken ill, have the disease in the mildest form; an instance of which came under our observation when we visited Fort Ramakins, and the same is confirmed by the experience of the natives.

The cloathing of the soldier should be of warm quality, and in the best repair; he should be equipped with woollen stockings and flannel waistcoats. The frequent changes of which merit particular attention from his officer. The shoes should be strong, of the best water proof leather, to guard against the peculiar damp of the country. The pantaloons should be of a spongy warm texture, blue or grey, in preference to white, that there may be no temptation to adopt the pernicious custom of cleaning them with wetted pipe clay.

The diet, especially in the sickly period, should be nutritive, and the broth well spiced with pepper; during which season, a small portion of unmixed spirit might be usefully allowed early in the morning.

Having ascertained that the crews of the vessels stationed in the very narrow channel (only a few yards from the land) between Beveland and Walcheren, have continued perfectly healthy the whole campaign; thus decidedly proveing that the noxious exhalation is nearly confined to its original source: We recommend, that before the commencement of the autumn of each year, several large ships be moored in convenient stations off the island, as floating barracks, to receive such part of the army as can be spared from the necessary garrison duties; and for the reception of the casual sick of that part of the army so embarked, a suitable number of hospital ships should be similarly anchored, to which also all convalescents on shore may be sent, who are recovering from the first attack of fever; by which early removal, fatal relapses so frequent among those who continue to be exposed to the local exhalations would effectually be prevented.

(Signed) J. BORLAND, M. D.

Inspector of Hospitals.

W. LEMPRIERE, M. D.

Physician to the Forces.

(Signed) G. BLANE, M. D.

